# Genetic Variation of *MLH1* (rs63749820) in Patients With Oral Squamous Cell Carcinoma

**DOI:** 10.1155/ijod/2710805

**Published:** 2026-05-18

**Authors:** Razieh Zare, Mohammad Javad Mokhtari, Hassan Rahimi, Mohammad Javad Fattahi, Bijan Khademi

**Affiliations:** ^1^ Department of Oral and Maxillofacial Pathology, School of Dentistry, Shiraz University of Medical Sciences, Shiraz, Iran, sums.ac.ir; ^2^ Department of Biology, Zarg.C., Islamic Azad University, Zarghan, Iran, zariau.ac.ir; ^3^ Student Research Committee, School of Dentistry, Shiraz University of Medical Sciences, Shiraz, Iran, sums.ac.ir; ^4^ Shiraz Institute for Cancer Research, School of Medicine, Shiraz University of Medical Sciences, Shiraz, Iran, sums.ac.ir; ^5^ Department of Otolaryngology, Khalili Hospital, Shiraz Institute for Cancer Research, Shiraz University of Medical Sciences, Shiraz, Iran, sums.ac.ir

**Keywords:** *MLH1*, OSCC, rs63749820, single nucleotide polymorphism

## Abstract

**Introduction:**

Oral squamous cell carcinoma (OSCC) is the most common type of cancer found in the oral cavity, exhibiting a persistent increase in incidence in developing nations. The mismatch repair (MMR) system preserves genomic stability during successive duplications. Mutator L Homolog 1 (MLH1) is a crucial component of the MMR and significantly contributes to mutation prevention. This study sought to evaluate the genetic variation of *MLH1* (rs63749820) in patients with OSCC.

**Methods:**

A total of 201 individuals were enrolled in the study, consisting of 101 patients diagnosed with OSCC and 100 control subjects. The *MLH1* variant rs63749820 was identified using tetra‐primer amplification‐refractory mutation system‐polymerase chain reaction (ARMS‐PCR).

**Results:**

Multivariate logistic regression analysis revealed that, within the codominant model, individuals carrying the TT genotype exhibited a significantly reduced risk of OSCC, with the odds ratio (OR) decreasing to 0.35 relative to those with the CC genotype (OR = 0.35, 95% CI = 0.15–0.81, and *p* = 0.01). In the prevailing model, the risk of OSCC was significantly reduced to 0.51‐fold in patients with TT + CT genotypes of rs63749820 compared to the CC genotype (OR = 0.48, 95% CI = 0.24–0.95, and *p* = 0.03). The T allele was notably less prevalent in OSCC patients (44.05%) than in controls (56%) (*p* = 0.01). No substantial differences were noted between clinicopathological characteristics and genotype.

**Conclusion:**

This research demonstrated a correlation between genetic variants in *MLH1* and the risk of OSCC within the Iranian population. The TT, rather than CT, is the key genotype conferring reduced susceptibility to OSCC in this population.

## 1. Introduction

Oral squamous cell carcinoma (OSCC) is the most common malignancy of the oral cavity and represents a major subset of head and neck squamous cell carcinoma (HNSCC) [1]. OSCC is the most aggressive neoplasm of oral malignancy, resulting in damage to the epithelial cells of the oral cavity due to the accumulation of numerous genetic mutations in these cells [2–5]. OSCC accounts for a significant proportion of cancer‐related morbidity and mortality worldwide, with increasing incidence particularly in developing countries [6]. Despite advances in diagnosis and treatment, OSCC remains aggressive and is associated with a poor prognosis.

Well‐established risk factors for OSCC encompass tobacco use, alcohol consumption, viral infections, and genetic predisposition [7]. Beyond environmental exposures, genetic susceptibility constitutes a pivotal determinant of individual risk [8]. Elucidating genetic variants that contribute to OSCC pathogenesis may enhance our understanding of underlying disease mechanisms and facilitate more precise risk stratification.

A variety of genetic mechanisms contribute to the establishment of a stable molecular microenvironment, among which the mismatch repair (MMR) pathway plays a central and indispensable role [9]. The MMR pathway is essential for maintaining genomic integrity by correcting base–base mismatches and insertion–deletion loops during DNA replication [10]. The eukaryotic MMR system comprises five mutator S (MutS) homologs, two mutator L (MutL) homologs, and three postmeiotic segregated proteins [11]. The *MLH1* gene, situated on chromosome 3p22.2, encodes a critical protein integral to the MMR pathway [12]. MLH1 not only prevents mutation accumulation but also mediates cell cycle arrest and apoptosis in response to DNA damage [13]. It has been demonstrated that MLH1 is crucial for the effective activation of caspase‐3 and caspase‐7, underscoring its pivotal role in initiating apoptosis through these key effector proteins. Moreover, evidence suggests that MLH1 contributes to the signaling pathways that lead to ataxia telangiectasia and Rad3 related (ATR), checkpoint kinase 1 (CHK1), and CHK2 activation [14]. In colon cancer cells, MLH1 has been reported to depend on ATR and ataxia‐telangiectasia mutated (ATM) to induce H2A histone family member X (H2AX) phosphorylation and cell cycle arrest while functioning independently of p53 in both processes [15, 16]. In recent years, MLH1 has garnered increasing research attention due to its established role as an essential constituent of the MMR pathway in microsatellite instability, with aberrations in its function being strongly associated with elevated cancer risk [17].

Single nucleotide polymorphisms (SNPs) in MMR genes represent irreversible alterations that can compromise the efficiency of the repair system. Consequently, the MMR machinery fails to correct base mismatches and insertion/deletion loops, leading to the accumulation of replication errors (RERs) and microsatellite instability during DNA synthesis [10]. Multiple polymorphisms were identified in the *MLH1* gene, some of which have been demonstrated to affect the expression of functional MLH1 [18]. *MLH1* promoter hypermethylation has been reported in 8%–69% of oral cancer specimens [19]. In hereditary cancers, somatic mutations primarily inactivate the *MLH1* gene, whereas in sporadic cancers, epigenetic silencing of *MLH1* through promoter hypermethylation is implicated [20]. In a prior report, the rs63749820 variant of *MLH1* correlated with breast cancer risk; however, it exhibited no correlation with overall survival or progression‐free survival. The brief duration of the follow‐up for study participants may account for the absence of correlation between *MLH1* polymorphisms and survival analysis. The two selected SNPs are clinically pathogenic; however, there is limited data regarding their association with cancer and protein structure prediction [21]. Additional research has highlighted the significance of MLH1 in various malignancies. In a case–control study involving 50 OSCC patients and 200 healthy controls, promoter methylation of *MLH1* was identified in 38 (76%) of the cancer cases, whereas it was absent in all control samples [22]. Czerninski et al. [23] illustrated that, within their cohort of OSCC, all patients with multiple oral malignancies exhibited hypermethylation of the *MLH1* promoter.

Dysfunction of MLH1 results in microsatellite instability, accumulation of mutations, and impaired apoptosis, all of which contribute to the pathogenesis of OSCC. The rs63749820 SNP has been reported to influence MLH1 expression and protein function in several malignancies, suggesting that it may alter DNA repair capacity and thereby modulate susceptibility to OSCC. Given the established role of MLH1 in carcinogenesis and the paucity of data regarding rs63749820 in oral cancers, this variant was selected as a biologically relevant biomarker to investigate its potential contribution to OSCC risk in the Iranian population. Although *MLH1* polymorphisms have been implicated in breast, colorectal, endometrial, and lung cancers, no prior study has evaluated the rs63749820 variant in OSCC. This gap in the literature provided the rationale for the present investigation, which aimed to assess the correlation between diverse clinicopathological factors and OSCC, as well as to analyze the rs63749820 SNP of the *MLH1* gene in both cases and controls. To our knowledge, this is the first study to explore the role of this *MLH1* variant in OSCC patients.

## 2. Materials and Methods

### 2.1. Study Subjects and Ethical Considerations

This case–control study was performed from April 2022 to July 2023, involving 101 patients diagnosed with new‐onset OSCC by two independent pathologists and 100 controls with no history of cancer or autoimmune disease. Participants in this study were selected from available samples from Namazi, Khalili, and Madar‐Va‐Kodak Hospitals affiliated with Shiraz University of Medical Sciences. The demographic characteristics of the samples are presented in Table 1. Healthy controls were recruited from individuals attending clinics in Fars Province for routine medical examinations during the same period. This study enrolled 101 patients with OSCC, aged 19–90 years, and 100 controls. The study received approval from the Medical Ethics Committee of Shiraz University of Medical Sciences, which assigned it the ethical code IR.SUMS.DENTAL.REC.1401.049. All individuals involved voluntarily agreed to take part in this experiment. Written informed consent was obtained from all participants before the commencement of the study. The criteria for inclusion and exclusion are detailed in Table 2.

**Table 1:** Demographic characteristics of the samples.

Samples	*N*	Gender		Mean age ± Std. deviation
Patient	101	Male	52	58.99 ± 14.69
		Female	49	
Control	100	Male	45	56.26 ± 13.43
		Female	55	

**Table 2:** Participant inclusion and exclusion criteria.

Inclusion (patient)	Exclusion (patient)
Initial diagnosis of SCC based on clinical signs and definitive diagnosis and based on pathology report	Patient unwillingness to participate in the study
Two pathologists confirmed the diagnosis of oral SCC	Simultaneous presence of other tumors
Determination of clinical staging of cases according to the tumor, node, and metastasis (TNM) staging system of the American Joint Committee on Cancer (AJCC)	Simultaneous presence of diseases with immunological and genetic backgrounds such as autoimmune disorders (AIDs) such as scleroderma, myositis, Sjogren syndrome, and lupus
Determination of the histopathological grade of oral SCC based on World Health Organization (WHO) criteria	—

**Inclusion (control)**	**Exclusion (control)**

No history of malignancy, clinical diseases such as oral diseases, or drug use	The presence or history of any type of cancer or autoimmune and genetic diseases in the individual or his/her first‐degree relatives
Physical health	—
No history of autoimmune diseases and no history of infectious diseases in the last month	—

### 2.2. Study Methods

Peripheral blood samples (~5 mL) were obtained from all participants using tubes containing ethylenediaminetetraacetic acid (EDTA) as an anticoagulant. Genomic DNA was extracted from whole blood samples using the salting‐out method, and DNA purity was subsequently evaluated by spectrophotometry with an Eppendorf BioPhotometer (Germany). The genomic sequence of *MLH1* was obtained from the National Center for Biotechnology Information (NCBI) website (http://www.ncbi.nlm.nih.gov). Primers were designed for tetra‐primer amplification‐refractory mutation system‐polymerase chain reaction (ARMS‐PCR), a straightforward and reliable method for SNP detection [24–26]. Table 3 displays the Tetra‐ARMS‐PCR primers utilized for SNPs in the *MLH1* gene. The PCR products were confirmed using 2% agarose gels. To ensure the quality of genotyping, ~20% of the randomly selected samples underwent sequencing, which confirmed the absence of genotyping errors. Negative control tubes containing the PCR mixture without DNA template were included in each run to monitor potential contamination.

**Table 3:** Primer sequences and PCR conditions related to the amplification of *MLH1* polymorphisms.

rs63749820	FI	CCCTCCTAAACCATGTGCTGGCACTT	26	95:5 min 95:45 S 70:45 S 72:55 S ^∗^30 cycles	220 bp (T allele)
	RI	ATTCTTACCGTGATCTGGGTCCCGTG	26		298 bp (C allele)
	FO	CTGGGAAACAAGAGCAAAACTCCGTCTC	28		467 bp
	RO	CCATGCCACAAAAGCCAATAGTCATTT	27		

### 2.3. Statistical Analysis

Descriptive statistics were utilized to analyze the general characteristics, clinicopathological information, and *MLH1* polymorphisms in patients with OSCC in comparison to the control group. Quantitative variables exhibited a normal distribution as determined by the Shapiro–Wilk test and were analyzed using *t*‐tests, while Chi‐square tests were employed for qualitative data. Conventional or ordinary logistic regression was utilized to evaluate the relationship between general demographic characteristics and *MLH1* variants as independent variables and OSCC as dependent variables. For this purpose, we computed the odds ratio (OR) along with the 95% confidence intervals (95% CIs) to evaluate the strength of the relationship between polymorphisms and the risk of OSCC. We set the threshold for statistical significance at *p*  < 0.05. We utilized SPSS software (version 27.0) for all analyses.

## 3. Results

### 3.1. General Characteristics

This case–control study included 101 OSCC cases with a mean age and SD of 58.99 ± 14.69. Among them, 49 were females (48.52%), and 52 were males (51.48%). The control group included 100 healthy community controls with a mean age and SD of 56.26 ± 13.43. Among them, 55 were females (55.00%), and 45 were males (45.00%). The distribution of genotypes indicated that the control group is in Hardy–Weinberg equilibrium (χ2 = 0.91, df = 1, and *p* = 0.33).

### 3.2. Genotyping of MLH1 rs63749820 Variant and the Risk of OSCC

Data analysis indicated that the CT genotype was the most prevalent in both cases (52.47%) and controls (54.00%), whereas the least frequent genotype was TT in cases (17.82%) and CC in controls (17.00%). Comparison of genotype and gender distributions between case and control groups revealed no statistically significant differences (*p*  > 0.05). The case and control groups were the same in terms of gender and genotype.

Table 4 presents the correlation between various genotypes of *MLH1* rs63749820 and the risk of OSCC in the Iranian population. The genotype distribution showed that CT was the most frequent genotype in both cases and controls, with very similar proportions, indicating no significant difference between groups. Allele frequency analysis, however, revealed that the T allele was less common in OSCC cases than in controls, suggesting a protective role (*p* = 0.01). In logistic regression, the TT genotype was associated with a significantly reduced risk of OSCC compared with the CC genotype, supporting its protective effect (OR = 0.35, 95% CI = 0.15–0.81, and *p* = 0.01). The dominant model (TT + CT vs. CC) also indicated a reduced risk of OSCC (OR = 0.48, 95% CI = 0.24–0.95, and *p* = 0.03); however, this association appears to be predominantly attributable to the TT genotype, since CT alone did not differ between groups. Individuals carrying at least one T allele (CT or TT genotypes) had a lower likelihood of developing OSCC compared to those with the CC genotype. These findings suggest that the T allele may confer a protective effect in both heterozygous and homozygous states.

**Table 4:** Distribution of genotypes and allele frequencies of *MLH1* rs63749820 gene polymorphism in the cases and control groups in different genetic models.

rs63749820	Patient group (%)	Control group (%)	OR (95% CI)	*p*‐Value
Codominant				
CC	30 (29.70)	17 (17.0)	1	—
TT	18 (17.82)	29 (29.0)	0.35 (0.15–0.81)	0.01
CT	53 (52.47)	54 (54.0)	0.55 (0.27–1.12)	0.10
Dominant				
CC	30 (29.70)	17 (17.0)	1	—
TT + CT	71 (70.29)	83 (83.0)	0.48 (0.24–0.95)	0.03
Recessive				
CC + CT	83 (82.17)	71 (71.0)	1	—
TT	18 (17.82)	29 (29.0)	0.53 (0.27–1.03)	0.06
Overdominant				
CC + TT	48 (47.52)	46 (46.0)	1	—
CT	53 (52.47)	54 (54.0)	0.94 (0.54–1.63)	0.82
Allele				
C	113 (55.94)	88 (44.0)	1	—
T	89 (44.05)	112 (56.0)	0.61 (0.41–0.91)	0.01

### 3.3. rs10757278 Polymorphism and Pathological Characteristics

Table 5 presents the clinical and pathological characteristics of the patients. The tumor stage was assessed using the TNM staging system, revealing that the majority of patients were classified as grade 1 (63.49%) and stage 3 (31.16%). Grade 4 and stage 1 were the least frequent. Among the patients, 55.07% had lymph node involvement. Data regarding tumor grade (38 patients), stage (24 patients), lymph node involvement (32 patients), and age (nine participants) were unavailable. The genotype of a patient who has grade 4 is CT (Table 5). No significant correlations were observed between genotype frequencies and clinicopathological parameters, including lymph node involvement (*p* = 0.937), grade (*p* = 0.850), stage (*p* = 0.303), tumor site (*p* = 0.882), and tumor size (*p* = 0.619). Also, no significant association was observed between genotype frequency and age (*p* = 0.613).

**Table 5:** Clinical and pathological characteristics of the patients.

Genotypes					
Variables		CC *N* (%)	CT *N* (%)	TT *N* (%)	*p*‐Value
Age (mean ± Std.deviation)		58.77 ± 15.67	58.38 ± 14.06	61.31 ± 15.10	0.613
Tumor size		4.62 ± 4.32	6.21 ± 8.06	6.76 ± 5.04	0.619
Tumor site	Oral^a^	8 (26.7)	16 (53.3)	6 (20)	0.882
	Tongue	22 (31)	37 (52.1)	12 (16.9)	
Grade	1	11 (27.5)	22 (55)	7 (17.5)	0.850
	2	6 (28.6)	10 (52.4)	4 (19)	
	3	0 (0)	2 (100)	0 (0)	
	4	0 (0)	1 (100)	0 (0)	
Stage	1	5 (35.7)	8 (57.1)	1 (7.1)	0.303
	2	7 (35)	12 (60)	1 (5)	
	3	7 (29.2)	14 (58.3)	3 (12.5)	
	4	6 (31.6)	7 (36.8)	6 (31.6)	
Lymph node involvement	+	11 (28.9)	20 (52.6)	7 (18.4)	0.937
	−	10 (32.3)	15 (48.4)	6 (19.4)	

^a^Oral (1 case of maxilla + 1 case of lip).

## 4. Discussion

This research aimed to ascertain the correlation between the *MLH1* SNP rs63749820 and the risk of OSCC in a southwestern Iranian population. We determined that rs63749820 is associated with the risk of OSCC. Moreover, no substantial correlation was observed between the frequency of *MLH1* genotypes and gender, lymph node involvement, tumor location, tumor classification, tumor dimensions, grade, and stage. This study represents the first examination of the correlation between *MLH1* rs63749820 polymorphisms and the risk of OSCC. In the multivariate logistic regression analysis, we discovered that the patients bearing the TT genotype compared to the CC genotype had a significantly lower OSCC risk in the codominant model. Furthermore, our analysis demonstrated a significantly reduced risk in patients carrying the TT + CT genotypes compared with those harboring the CC genotype under the dominant model. Although the CT genotype appeared similarly distributed among cases and controls, the dominant model analysis revealed a significant protective association for individuals carrying at least one T allele. This apparent discrepancy can be attributed to the nature of the dominant model, which evaluates the combined effect of TT and CT genotypes relative to CC. While CT alone may not show a pronounced effect in isolation, its inclusion alongside TT may reflect a cumulative influence. These findings underscore the importance of considering genetic models that account for allele interactions rather than relying solely on raw genotype frequencies. However, given the lack of difference in CT genotype frequency between groups, it is likely that the observed effect in the dominant model is primarily attributable to the TT genotype. Further studies with larger cohorts and stratified analyses are warranted to clarify the individual contributions of *MLH1* genotypes to OSCC susceptibility. In earlier research, we assessed the relationship between polymorphisms of various genes and OSCC [5, 27, 28].

MLH1 is a highly expressed MMR protein, and its functionality is crucial in this pathway, resulting in pathological consequences when impaired. Multiple genetic mutations in the *MLH1* gene contribute to breast cancer. Genetic variants are categorized into various classifications according to their function and clinical relevance. Several studies have reported associations between *MLH1* polymorphisms and susceptibility to lung, endometrial, and colorectal cancers [29, 30]. The SNP examined in this research is considered clinically pathogenic [21]; however, there is insufficient data regarding its correlation with cancer. SNPs were detected in nearly all MMR genes, primarily in *MLH1* and *MSH2* [31].

In a meta‐analysis of 17 studies, Pan et al. [32] found no association between the *MLH1* SNP rs1800734 and an increased risk of cancer. However, subsequent research aligned with our findings, revealing a significant relationship between *MLH1* variants and cancer susceptibility. Malik et al. [21] reported that *MLH1* polymorphisms rs63749795 and rs63749820 were significantly associated with a higher risk of breast cancer. Their study also noted downregulation of *MLH1* gene expression and loss of MLH1 protein in breast cancer cases, suggesting its role in disease progression. Notably, the absence of MLH1 protein correlated with higher‐grade tumors and lymph node positivity, highlighting its importance in the MMR system [21]. Dreussi et al. [33] identified a protective effect of *MLH1* SNP rs1799977 in rectal cancer patients.

Lin et al. [34] identified a weak linkage disequilibrium between the *MLH1* rs1800734 and rs1540354 polymorphisms. Their findings indicated that OSCC patients harboring the rs1800734 AA genotype exhibited significantly poorer prognoses. This SNP may also predict treatment outcomes in patients with OSCC undergoing postoperative adjuvant radiotherapy, particularly in advanced stages. In contrast, no statistically significant differences in clinical outcomes were observed for the rs1540354 genotypes [34]. Moreover, another investigation reported a correlation between the *MLH1*‐93 A > G polymorphism and an elevated risk of tobacco‐related OSCC in Asian Indian populations, suggesting its potential utility in screening high‐risk groups [35]. Zare et al. [36] found that the *MLH1* rs63749795 variant may be associated with reduced OSCC susceptibility under the dominant genetic model. Furthermore, Senghore et al. [37] reported that the GG genotype of *MLH1* rs1800734 is associated with improved survival rates in patients with OSCC treated with adjuvant concurrent chemoradiotherapy. These genetic polymorphisms may therefore represent valuable prognostic biomarkers in the management of OSCC. Fernandes et al. [38] found no significant variation in MLH1 expression across different histopathological grades of OSCC. Similarly, Chaudhari et al. [39] found no correlation between MLH1 immunoexpression and demographic variables, including gender, age, or sample size, across control, leukoplakia, and OSCC groups. Other studies have demonstrated that microsatellite instability in OSCC samples is significantly associated with tumor stage, histological differentiation grade, and an increased risk of developing multiple oral malignancies [23, 40, 41]. Discrepancies between these studies and our findings could be attributed to limited sample sizes and incomplete patient data.

The current study faces several limitations, including the diverse ethnic composition of the southwestern Iranian population and a small sample size, both of which may have influenced the findings. These limitations highlight the need for future investigations to incorporate larger, ethnically diverse cohorts and employ statistical methods specifically designed to detect and control for population structure in genetic association studies. One major constraint is the lack of complete clinicopathological data—specifically tumor grade, disease stage, and lymph node status—which are crucial for adequately analyzing genotype–phenotype associations with statistical reliability. The absence of this information could weaken the robustness of the results and obscure meaningful correlations. Furthermore, information was collected based on patients’ self‐reports in the forms, and patients had incomplete information regarding variables known to influence OSCC risk. Because we used samples available at the center in our sampling method, an atypical gender distribution was observed in the patients. Future research should pursue prospective data collection, more thorough clinicopathological profiling, and culturally sensitive methods to measure confounding variables, thereby improving both the accuracy and generalizability of findings.

## 5. Conclusion

This study is the first to demonstrate an association between *MLH1* rs63749820 and the risk of OSCC, thereby advancing current understanding of genetic susceptibility in oral cancers. We identified that rs63749820 is correlated with the risk of OSCC. The protective effects of TT against OSCC were likewise evidenced. No notable differences were detected between clinicopathological characteristics and genotype. A meta‐analysis of studies conducted across diverse regions, complemented by independent case–control investigations, is required to clarify the role of *MLH1* polymorphisms in OSCC.

## Author Contributions

Razieh Zare, Hassan Rahimi, and Mohammad Javad Mokhtari conceived and planned the presented experiment. Razieh Zare, Hassan Rahimi, Mohammad Javad Mokhtari, Mohammad Javad Fattahi, and Bijan Khademi performed experiments and analyzed the data. Razieh Zare and Mohammad Javad Mokhtari supervised the research, designed experiments, and wrote the paper.

## Funding

This study was funded by the Research Vice‐Chancellery of Shiraz University of Medical Sciences (Grant 24913).

## Disclosure

All authors have read and approved the manuscript.

## Ethics Statement

This study was reviewed and approved by the Medical Ethics Committee of Shiraz University of Medical Sciences, under Approval Number IR.SUMS.DENTAL.REC.1401.049. All procedures involving human participants were conducted in accordance with the ethical standards of the institutional and/or national research committee and with the 1964 Helsinki Declaration and its later amendments.

## Conflicts of Interest

The authors declare no conflicts of interest.

## Data Availability

The data that support the findings of this study are available from the corresponding author upon reasonable request.

## References

[1] Tan Y. , Wang Z. , and Xu M. , et al.Oral Squamous Cell Carcinomas: State of the Field and Emerging Directions, International Journal of Oral Science. (2023) 15, no. 1, 10.1038/s41368-023-00249-w, 44. 37736748 PMC10517027

[2] Bagan J. , Sarrion G. , and Jimenez Y. , Oral Cancer: Clinical Features, Oral Oncology. (2010) 46, no. 6, 414–417, 10.1016/j.oraloncology.2010.03.009, 2-s2.0-77952743003. 20400366

[3] Gharat S. A. , Momin M. M. , and Bhavsar C. , Oral Squamous Cell Carcinoma: Current Treatment Strategies and Nanotechnology-Based Approaches for Prevention and Therapy, Critical Reviews in Therapeutic Drug Carrier Systems. (2016) 33, no. 4, 363–400, 10.1615/CritRevTherDrugCarrierSyst.2016016272, 2-s2.0-85013223158. 27910740

[4] Neville B. W. and Day T. A. , Oral Cancer and Precancerous Lesions, CA: A Cancer Journal for Clinicians. (2002) 52, no. 4, 195–215, 10.3322/canjclin.52.4.195, 2-s2.0-0036347157. 12139232

[5] Ghapanchi J. , Ranjbar Z. , and Mokhtari M. J. , et al.The *LncRNA H19* rs217727 Polymorphism Is Associated With Oral Squamous Cell Carcinoma Susceptibility in Iranian Population, BioMed Research International. (2020) 2020, no. 1, 10.1155/2020/1634252, 1634252. 32337223 PMC7154967

[6] Fatima J. , Fatima E. , and Mehmood F. , et al.Comprehensive Analysis of Oral Squamous Cell Carcinomas: Clinical, Epidemiological, and Histopathological Insights With a Focus on Prognostic Factors and Survival Time, Cureus. (2024) 16, 10.7759/cureus.54394. PMC10949903 38505442

[7] Shebbo S. , Alateyah N. , and Yassin E. , et al.Unravelling Molecular Mechanism of Oral Squamous Cell Carcinoma and Genetic Landscape: An Insight Into Disease Complexity, Available Therapies, and Future Considerations, Frontiers in Immunology. (2025) 16, 10.3389/fimmu.2025.1626243, 1626243. 40881676 PMC12380768

[8] Jumparway D. , Su C.-W. , and Yen A. M.-F. , et al.Quantifying Genetic and Environmental Factors Accounting for Multistage Progression of Precancerous Lesions and Oral Cancer: Applications to Risk-Guided Prevention, Cancers. (2025) 17, no. 13, 10.3390/cancers17132114, 2114. 40647413 PMC12248456

[9] Pećina-Šlaus N. , Kafka A. , Salamon I. , and Bukovac A. , Mismatch Repair Pathway, Genome Stability and Cancer, Frontiers in Molecular Biosciences. (2020) 7, 10.3389/fmolb.2020.00122, 122. 32671096 PMC7332687

[10] He Y. , Zhang L. , Zhou R. , Wang Y. , and Chen H. , The Role of DNA Mismatch Repair in Immunotherapy of Human Cancer, International Journal of Biological Sciences. (2022) 18, no. 7, 2821–2832, 10.7150/ijbs.71714. 35541922 PMC9066103

[11] Kunkel T. A. and Erie D. A. , Eukaryotic Mismatch Repair in Relation to DNA Replication, Annual Review of Genetics. (2015) 49, no. 1, 291–313, 10.1146/annurev-genet-112414-054722, 2-s2.0-84948741378. PMC5439269 26436461

[12] Wong J. J. , Hawkins N. J. , Ward R. L. , and Hitchins M. P. , Methylation of the 3p22 Region Encompassing MLH1 Is Representative of the CpG Island Methylator Phenotype in Colorectal Cancer, Modern Pathology. (2011) 24, no. 3, 396–411, 10.1038/modpathol.2010.212, 2-s2.0-79952191728. 21102416

[13] Gupta D. and Heinen C. D. , The Mismatch Repair-Dependent DNA Damage Response: Mechanisms and Implications, DNA Repair. (2019) 78, 60–69, 10.1016/j.dnarep.2019.03.009, 2-s2.0-85063905380. 30959407

[14] Wu Q. and Vasquez K. M. , Human MLH1 Protein Participates in Genomic Damage Checkpoint Signaling in Response to DNA Interstrand Crosslinks, While MSH2 Functions in DNA Repair, PLoS Genetics. (2008) 4, no. 9, 10.1371/journal.pgen.1000189, 2-s2.0-52949152426, e1000189. 18787700 PMC2526179

[15] Cejka P. , Stojic L. , and Mojas N. , et al.Methylation-Induced G2/M Arrest Requires a Full Complement of the Mismatch Repair Protein hMLH1, The EMBO Journal. (2003) 22, no. 9, 2245–2254. 12727890 10.1093/emboj/cdg216 PMC156088

[16] McDaid J. , Loughery J. , and Dunne P. , et al.MLH1 Mediates PARP-Dependent Cell Death in Response to the Methylating Agent N-Methyl-N-Nitrosourea, British Journal of Cancer. (2009) 101, no. 3, 441–451, 10.1038/sj.bjc.6605186, 2-s2.0-68149137729. 19623177 PMC2720233

[17] Awosika J. A. , Gulley J. L. , and Pastor D. M. , Deficient Mismatch Repair and Microsatellite Instability in Solid Tumors, International Journal of Molecular Sciences. (2025) 26, no. 9, 10.3390/ijms26094394, 4394. 40362635 PMC12072705

[18] Li S. , Zheng Y. , and Tian T. , et al.Pooling-Analysis on hMLH1 Polymorphisms and Cancer Risk: Evidence Based on 31,484 Cancer Cases and 45,494 Cancer-Free Controls, Oncotarget. (2017) 8, no. 54, 93063–93078, 10.18632/oncotarget.21810, 2-s2.0-85032699740. 29190978 PMC5696244

[19] Jenkins G. , O’Byrne K. J. , Panizza B. , and Richard D. J. , Genome Stability Pathways in Head and Neck Cancers, International Journal of Genomics. (2013) 2013, 10.1155/2013/464720, 2-s2.0-84889016853, 464720. 24364026 PMC3834617

[20] Kuismanen S. A. , Holmberg M. T. , Salovaara R. , de la Chapelle A. , and Peltomäki P. , Genetic and Epigenetic Modification of MLH1 Accounts for a Major Share of Microsatellite-Unstable Colorectal Cancers, The American Journal of Pathology. (2000) 156, no. 5, 1773–1779, 10.1016/S0002-9440(10)65048-1, 2-s2.0-0033847512. 10793088 PMC1876911

[21] Malik S. S. , Zia A. , and Mubarik S. , et al.Correlation of MLH1 Polymorphisms, Survival Statistics, in Silico Assessment and Gene Downregulation With Clinical Outcomes Among Breast Cancer Cases, Molecular Biology Reports. (2020) 47, no. 1, 683–692, 10.1007/s11033-019-05175-x. 31701475

[22] González-Ramírez I. , Ramirez-Amador V. , and Irigoyen-Camacho M. , et al.hMLH1 Promoter Methylation Is an Early Event in Oral Cancer, Oral Oncology. (2011) 47, no. 1, 22–26, 10.1016/j.oraloncology.2010.10.002, 2-s2.0-79551536814. 21075045

[23] Czerninski R. , Krichevsky S. , Ashhab Y. , Gazit D. , Patel V. , and Ben-Yehuda D. , Promoter Hypermethylation of Mismatch Repair Genes, hMLH1 and hMSH2 in Oral Squamous Cell Carcinoma, Oral Diseases. (2009) 15, no. 3, 206–213, 10.1111/j.1601-0825.2008.01510.x, 2-s2.0-62749188773. 19207881

[24] Randhawa R. , Duseja A. , and Changotra H. , A Novel Tetra-Primer ARMS-PCR Based Assay for Genotyping SNP rs12303764(G/T) of Human Unc-51 like kinase 1 Gene, Molecular Biology Reports. (2017) 44, no. 1, 1–4, 10.1007/s11033-016-4087-7, 2-s2.0-84992411952. 27783190

[25] Sadeghi Moghadam F. , Mokhtari M. J. , and Fattahi M. J. , LncRNA H19 Genotypes and Haplotypes in Ovarian Cancer Patients, Journal of Obstetrics, Gynecology and Cancer Research. (2024) 10, no. 6, 468–474.

[26] Sadeghi Z. and Mokhtari M. J. , Association of rs12826786 and rs1899663, Long Non-Coding RNA HOTAIR Polymorphic Variants, With Susceptibility to Rheumatoid Arthritis Disease, Indian Journal of Clinical Biochemistry. (2024) 1–8, 10.1007/s12291-024-01238-4. PMC12988054 41834934

[27] Salah M. , Rezaee M. , Fattahi M. J. , Ghaderi A. , Khademi B. , and Mokhtari M. J. , Association of lncRNA ANRIL rs10757278 A>G Variant, Tumor Size, Grading, Tumor Site, and Tumor Stage in Oral Squamous Cell Carcinoma Patients, Reports of Biochemistry and Molecular Biology. (2024) 13, no. 1, 59–66, 10.61186/rbmb.13.1.59. 39582818 PMC11580132

[28] Amiri M. A. , Amiri D. , and Mokhtari M. J. , et al.Allelic and Genotypic Analysis of LncRNA ANRIL rs4977574 A/G Mutations in Oral Squamous Cell Carcinoma Patients: Insights Into Tumor Characteristics and Genotypic Correlations, International Journal of Dentistry. (2023) 2023, 10.1155/2023/7738719, 7738719. 37829275 PMC10567505

[29] Ma Y. , Chen Y. , and Petersen I. , Expression and Promoter DNA Methylation of MLH1 in Colorectal Cancer and Lung Cancer, Pathology-Research and Practice. (2017) 213, no. 4, 333–338, 10.1016/j.prp.2017.01.014, 2-s2.0-85012907591. 28214209

[30] Buchanan D. D. , Tan Y. Y. , and Walsh M. D. , et al.Tumor Mismatch Repair Immunohistochemistry and DNA MLH1 Methylation Testing of Patients With Endometrial Cancer Diagnosed at Age Younger Than 60 Years Optimizes Triage for Population-Level Germline Mismatch Repair Gene Mutation Testing, Journal of Clinical Oncology. (2014) 32, no. 2, 90–100, 10.1200/JCO.2013.51.2129, 2-s2.0-84897018139. 24323032 PMC4876359

[31] Caja F. , Vodickova L. , Kral J. , Vymetalkova V. , Naccarati A. , and Vodicka P. , DNA Mismatch Repair Gene Variants in Sporadic Solid Cancers, International Journal of Molecular Sciences. (2020) 21, no. 15, 10.3390/ijms21155561, 5561. 32756484 PMC7432688

[32] Pan X.-M. , Yang W.-Z. , and Xu G.-H. , et al.The Association Between MLH1-93 G> A Polymorphism of DNA Mismatch Repair and Cancer Susceptibility: A Meta-Analysis, Mutagenesis. (2011) 26, no. 5, 667–673, 10.1093/mutage/ger032, 2-s2.0-80052329093. 21745804

[33] Dreussi E. , Cecchin E. , and Polesel J. , et al.Pharmacogenetics Biomarkers and Their Specific Role in Neoadjuvant Chemoradiotherapy Treatments: An Exploratory Study on Rectal Cancer Patients, International Journal of Molecular Sciences. (2016) 17, no. 9, 10.3390/ijms17091482, 2-s2.0-84985942521, 1482. 27608007 PMC5037760

[34] Lin L.-H. , Lin M.-W. , and Mar K. , et al.The hMLH1− 93G> A Promoter Polymorphism Is Associates With Outcomes in Oral Squamous Cell Carcinoma Patients, Annals of Surgical Oncology. (2014) 21, no. 13, 4270–4277, 10.1245/s10434-014-3897-x, 2-s2.0-84911959680. 25047469

[35] Jha R. , Gaur P. , Sharma S. C. , and Das S. N. , Single Nucleotide Polymorphism in hMLH1 Promoter and Risk of Tobacco-Related Oral Carcinoma in High-Risk Asian Indians, Gene. (2013) 526, no. 2, 223–227, 10.1016/j.gene.2013.05.014, 2-s2.0-84880749914. 23727610

[36] Zare R. , Mokhtari M. J. , Fattahi M. J. , Mohammadi N. , and Khademi B. , Reduced Oral Squamous Cell Carcinoma (2025) Risk Associated With MLH1 rs63749795 Polymorphism in the Dominant Genetic Model, Reports of Biochemistry and Molecular Biology. (2025) 14, no. 1, 215–222.

[37] Senghore T. , Wang W.-C. , and Chien H.-T. , et al.Polymorphisms of Mismatch Repair Pathway Genes Predict Clinical Outcomes in Oral Squamous Cell Carcinoma Patients Receiving Adjuvant Concurrent Chemoradiotherapy, Cancers. (2019) 11, no. 5, 10.3390/cancers11050598, 2-s2.0-85068437581, 598. 31035658 PMC6562473

[38] Fernandes A. M. , Ramos-Jorge M. L. , Cardoso S. V. , Loyola A. M. , Mesquita R. A. , and Aguiar M. C. F. , Immunoexpression of hMSH2 and hMLH1 in Oral Squamous Cell Carcinoma and Its Relationship to Histological Grades of malignancy, Journal of Oral Pathology & Medicine. (2008) 37, no. 9, 543–548, 10.1111/j.1600-0714.2008.00658.x, 2-s2.0-51349113745. 18331286

[39] Chaudhari N. T. , Tupkari J. V. , Joy T. , and Ahire M. S. , Human MutL Homolog 1 Immunoexpression in Oral Leukoplakia and Oral Squamous Cell Carcinoma: A Prospective Study in Indian Population, Journal of Oral and Maxillofacial Pathology. (2016) 20, no. 3, 453–461, 10.4103/0973-029X.190948, 2-s2.0-84988699071. 27721611 PMC5051294

[40] Christensen M. , Jensen M. A. , Wolf H. , and Ørntoft T. F. , Pronounced Microsatellite Instability in Transitional Cell Carcinomas From Young Patients With Bladder Cancer, International Journal of Cancer. (1998) 79, no. 4, 396–401. 9699533 10.1002/(sici)1097-0215(19980821)79:4<396::aid-ijc15>3.0.co;2-3

[41] Ashazila M. J. , Kannan T. , Venkatesh R. , and Hoh B. , Microsatellite Instability and Loss of Heterozygosity in Oral Squamous Cell Carcinoma in Malaysian Population, Oral Oncology. (2011) 47, no. 5, 358–364, 10.1016/j.oraloncology.2011.03.005, 2-s2.0-79956218338. 21450513

